# Surgical Experience and Functional Outcomes after Laparoscopic and Robot-Assisted Partial Nephrectomy: Results from a Multi-Institutional Collaboration

**DOI:** 10.3390/jcm13196016

**Published:** 2024-10-09

**Authors:** Carlo Andrea Bravi, Paolo Dell’Oglio, Angela Pecoraro, Zine-Eddine Khene, Riccardo Campi, Pietro Diana, Chiara Re, Carlo Giulioni, Alp Tuna Beksac, Riccardo Bertolo, Tarek Ajami, Kennedy Okhawere, Margaret Meagher, Arman Alimohammadi, Marco Borghesi, Andrea Mari, Daniele Amparore, Marco Roscigno, Umberto Anceschi, Giuseppe Simone, Nazareno Suardi, Antonio Galfano, Riccardo Schiavina, Federico Dehò, Karim Bensalah, Abdullah Erdem Canda, Vincenzo Ferrara, Antonio Alcaraz, Xu Zhang, Carlo Terrone, Shahrokh Shariat, Francesco Porpiglia, Alessandro Antonelli, Jihad Kaouk, Ketan Badani, Andrea Minervini, Ithaar Derweesh, Alberto Breda, Alexandre Mottrie, Francesco Montorsi, Alessandro Larcher

**Affiliations:** 1 Department of Urology, The Royal Marsden NHS Foundation Trust, London SW3 6JJ, UK; 2 Department of Urology, Onze-Lieve-Vrouwziekenhuis Hospital, 9300 Aalst, Belgium; a.mottrie@gmail.com; 3 ORSI Academy, 9090 Ghent, Belgium; 4 Unit of Urology, Division of Oncology, URI, IRCCS Ospedale San Raffaele, 20132 Milan, Italy; chiarare55@gmail.com (C.R.); montorsi.francesco@hsr.it (F.M.); larcher.alessandro@hsr.it (A.L.); 5 Department of Urology, ASST Grande Ospedale Metropolitano Niguarda, 20162 Milan, Italy; paolo.delloglio@gmail.com (P.D.); antoniogalfano@gmail.com (A.G.); 6 Department of Urology, Antoni van Leeuwenhoek Hospital, The Netherlands Cancer Institute, 1066 CX Amsterdam, The Netherlands; 7 Interventional Molecular Imaging Laboratory, Department of Radiology, Leiden University Medical Center, 2333 ZA Leiden, The Netherlands; 8 Department of Urology, Hospital Pederzoli, Peschiera del Garda, 37019 Verona, Italy; 9 Department of Urology, University of Rennes, 35700 Rennes, France; khene.zineddine@gmail.com (Z.-E.K.); karimbensalah@yahoo.fr (K.B.); 10 Unit of Urological Robotic Surgery and Renal Transplantation, Department of Experimental and Clinical Medicine, Careggi Hospital, University of Florence, 50134 Florence, Italy; riccardo.campi@gmail.com; 11 Department of Urology, Fundació Puigvert, Autonoma University of Barcelona, 08025 Barcelona, Spain; pietros.diana@gmail.com (P.D.); albbred@gmail.com (A.B.); 12 ASST-Sette Laghi, Circolo & Fondazione Macchi Hospital, University of Insubria, 21100 Varese, Italy; deho.federico@gmail.com; 13 Unit of Urology, Jesi Hospital, Jesi, 60035 Ancona, Italy; carlo.giulioni9@gmail.com (C.G.); vincenzoferrara4@gmail.com (V.F.); 14 Department of Urology, Polytechnic University of Marche Region, 60121 Ancona, Italy; 15 Glickman Urological & Kidney Institute, Cleveland Clinic, Cleveland, OH 44106, USA; beksaca@ccf.org (A.T.B.); kaoukj@ccf.org (J.K.); 16 Department of Urology, San Carlo Di Nancy Hospital, 00165 Rome, Italy; riccardobertolo@hotmail.it; 17 Department of Urology, Hospital Clinic-IDIBAPS, University of Barcelona, 08036 Barcelona, Spain; ajami@clinic.cat (T.A.); aalcaraz@clinic.cat (A.A.); 18 Department of Urology, Icahn School of Medicine at Mount Sinai, New York City, NY 10029, USA; kennedy.okhawere@mountsinai.org (K.O.); ketan.badani@mountsinai.org (K.B.); 19 Department of Urology, University of California, La Jolla, San Diego, CA 92103, USA; mfmeaghe@health.ucsd.edu (M.M.); iderweesh@health.ucsd.edu (I.D.); 20 Department of Urology, Medical University of Vienna, 1090 Vienna, Austria; armanalimohammadi@hotmail.com (A.A.); sfshariat@gmail.com (S.S.); 21 Department of Surgical and Diagnostic Integrated Sciences (DISC), University of Genova, 16132 Genova, Italy; mark.borghesi1@gmail.com (M.B.); carlo.terrone@med.uniupo.it (C.T.); 22 Unit of Oncologic Minimally-Invasive Urology and Andrology—Careggi Hospital, Department of Clinical and Experimental Medicine, University of Florence, 50134 Florence, Italy; andreamari08@gmail.com (A.M.); andrea.minervini@unifi.it (A.M.); 23 Department of Oncology, Division of Urology, University of Turin, San Luigi Gonzaga Hospital, Orbassano, 10043 Turin, Italy; danieleamparore@hotmail.it (D.A.); porpiglia@libero.it (F.P.); 24 ASST Papa Giovanni XXIII, 24125 Bergamo, Italy; roscigno.marco@gmail.com; 25 School of Medicine, University of Milano-Bicocca, 20132 Milan, Italy; 26 Department of Urology, IRCCS “Regina Elena” National Cancer Institute, 00128 Rome, Italy; umberto.anceschi@gmail.com (U.A.); puldet@gmail.com (G.S.); 27 Department of Urology, University of Brescia, 25123 Brescia, Italy; suardi.nazareno@gmail.com; 28 Division of Urology, IRCCS Azienda Ospedaliero-Universitaria di Bologna, 40138 Bologna, Italy; rschiavina@yahoo.it; 29 Department of Urology, Koç University Hospital, Istanbul 34010, Turkey; erdemcanda@yahoo.com; 30 RMK AIMES, Rahmi M. Koç Academy of Interventional Medicine, Education, and Simulation, Istanbul 34010, Turkey; 31 Department of Urology, Chinese PLA General Hospital, Beijing 100091, China; xzhang@foxmail.com; 32 Institute for Urology and Reproductive Health, Sechenov University, 119435 Moscow, Russia; 33 Department of Urology, Azienda Ospedaliera Universitaria Integrata, University of Verona, 37126 Verona, Italy; alessandro_antonelli@me.com

**Keywords:** partial nephrectomy, functional outcomes, acute kidney injury, robot-assisted surgery, learning curve, surgical experience

## Abstract

**Background:** In patients treated with partial nephrectomy, prior evidence showed that peri-operative outcomes, such as complications and ischemia time, improved as a function of the surgical experience of the surgeon, but data on functional outcomes after surgery are still scarce. **Methods:** We retrospectively analyzed data of 4011 patients with a single, unilateral cT1a-b renal mass treated with laparoscopic or robot-assisted partial nephrectomy. The operations were performed by 119 surgeons at 22 participating institutions between 1997 and 2022. Multivariable models investigated the association between surgical experience (number of prior operations) and acute kidney injury (AKI) and recovery of at least 90% of baseline estimated glomerular filtration rate (eGFR) 1 yr after partial nephrectomy. The adjustment for case mix included age, Body Mass Index, preoperative serum creatinine, clinical T stage, PADUA score, warm ischemia time, pathologic tumor size, and year of surgery. **Results:** A total of 753 (19%) and 3258 (81%) patients underwent laparoscopic and robot-assisted partial nephrectomy, respectively. Overall, 37 (31%) and 55 (46%) surgeons contributed only to laparoscopic and robotic learning curves, respectively, whereas 27 (23%) contributed to the learning curves of both approaches. In the laparoscopic group, 8% and 55% of patients developed AKI and recovered at least 90% of their baseline eGFR, respectively. After adjusting for confounders, we did not find evidence of an association between surgical experience and AKI after laparoscopic partial nephrectomy (odds ratio [OR]: 0.9992; 95% confidence interval [CI]: 0.9963, 1.0022; *p* = 0.6). Similar results were found when 1-year renal function was the outcome of interest (OR: 0.9996; 95% CI: 0.9988, 1.0005; *p* = 0.5). Among patients who underwent robot-assisted partial nephrectomy, AKI occurred in 11% of patients, whereas 54% recovered at least 90% of their baseline eGFR. On multivariable analyses, the relationship between surgical experience and AKI after surgery was not statistically significant (OR: 1.0015; 95% CI: 0.9992, 1.0037; *p* = 0.2), with similar results when the outcome of interest was renal function one year after surgery (OR: 1.0001; 95% CI: 0.9980, 1.0022; *p* = 0.9). Virtually the same findings were found on sensitivity analyses. **Conclusions:** In patients treated with laparoscopic or robot-assisted partial nephrectomy, our data suggest that the surgical experience of the operating surgeon might not be a key determinant of functional recovery after surgery. This raises questions about the use of serum markers to assess functional recovery in patients with two kidneys and opens the discussion on what are the key steps of the procedure that allowed surgeons to achieve optimal outcomes since their initial cases.

## 1. Introduction

The importance of laparoscopic partial nephrectomy and robot-assisted partial nephrectomy (RAPN) in modern surgical practice cannot be overstated. These minimally invasive techniques offer significant advantages over traditional open surgery [1]. They typically result in smaller incisions, reduced blood loss, less post-operative pain, and shorter hospital stays. Additionally, the precision afforded by robotic assistance can enhance the surgeon’s ability to perform delicate dissections and reconstructions, potentially preserving more of the healthy renal tissue and thus maintaining better overall kidney function. The technological advancements in these procedures also facilitate improved visualization of the surgical field, allowing for more accurate and controlled removal of renal tumors. This is particularly beneficial in complex cases where the tumor’s location and the patient’s anatomy present challenges. Consequently, laparoscopic partial nephrectomy and RAPN have become the preferred methods for treating localized kidney tumors, offering patients faster recovery times and better functional outcomes compared to conventional open surgery.

The learning curve for partial nephrectomy represents the process of gaining expertise in performing the surgical procedure over time. It takes considerable time and practice to develop the necessary skills to perform a successful partial nephrectomy with minimal complications [2]. Evidence from prior studies indicates that peri-operative outcomes, including complications and ischemia time, improved with increased surgical experience [2], but data on the potential impact of surgeon experience on functional outcomes after surgery are still scarce. In particular, a relationship has been established between acute kidney injury (AKI), a sudden episode of kidney failure that might happen after kidney surgery, and long-term consequences such as chronic kidney disease [3]. This underscores the importance of early intervention, careful monitoring, and appropriate management strategies to mitigate the risk of long-term renal impairment.

Given the complexity of partial nephrectomy, understanding the nuances of the learning curve is crucial for improving patient outcomes. Surgeons gradually enhance their technical skills and decision-making abilities through repeated practice and exposure to various surgical scenarios. This gradual improvement has been documented to lead to better peri-operative outcomes, highlighting the importance of experience. In this context, it has been demonstrated that early functional impairment after surgery—i.e., AKI—is correlated with long-term renal function after partial nephrectomy [3,4]; however, whether surgical experience might affect both AKI and long-term function after partial nephrectomy remains unknown. For this reason, we relied on a multi-institutional database including multiple surgeons to investigate the association between surgical experience and functional outcomes in patients undergoing laparoscopic partial nephrectomy and robot-assisted partial nephrectomy (RAPN).

## 2. Methods

### 2.1. Patient Population

We retrospectively analyzed the data of 8850 patients with a single, unilateral cT1_a-b_N0M0 renal mass treated with laparoscopic or robot-assisted partial nephrectomy. Operations were performed at twenty-two participating institutions between 1997 and 2022. Patients who had missing data for covariates (*n* = 3223) were excluded, leaving 5626 patients eligible for the analyses (a full description is provided in Supplementary Figure S1).

For the scope of this study, we focused on patients with two kidneys, aged 30–80 with a PADUA 6–9 renal mass and a preoperative estimated glomerular filtration rate (eGFR) greater than 45 mL/min/1.73 m^2^ (*n* = 4011). Given the heterogeneity of a complex procedure such as partial nephrectomy, the aim of these inclusion criteria was to limit confounding and to create a cohort of patients as homogeneous as possible, in order to test our hypothesis of an association between surgical experience and functional outcomes after surgery. All information was obtained with appropriate ethics committee or institutional review board waivers, and the data were anonymized before analysis.

Eligible patients were treated by one of 119 surgeons. Surgeons who had previously performed partial nephrectomy before their first procedure on a patient in the study cohort were asked to provide details of their previous approach-specific case load. Most surgeons performed their cases at the same institution, whereas 3 surgeons reported having moved between two institutions included in the study.

### 2.2. Outcome Definition

Our primary goal was to investigate the association between surgical experience and functional outcomes after partial nephrectomy, namely acute kidney injury and recovery of at least 90% of baseline eGFR 1 yr after partial nephrectomy [5]. Acute kidney injury was defined according to the KDIGO criteria [6]. Renal function at 1 yr follow-up was calculated using any eGFR measurement in the window between 9 and 15 months after partial nephrectomy. In case of multiple measurements, we considered the one closest to the 1 yr landmark.

### 2.3. Statistical Analyses

Our statistical analyses involved several steps [7]. According to prior methodology [8], surgeon experience was coded as the number of laparoscopic/robotic partial nephrectomies performed by the surgeon before the index patient’s operation [2,9,10,11]. Since the surgical approach may influence the outcomes of surgery [12,13], surgical experience was calculated in an approach-specific manner; in other words, the number of, say, laparoscopic cases performed before a certain index robotic operation did not count towards a given surgeon’s robotic experience, and vice versa. Surgeon experience was entered as a continuous variable, using restricted cubic splines with knots at the tertiles to create a non-linear relationship between experience and the outcomes of interest.

We assessed the association between a surgeon’s experience and acute kidney injury and 1 yr renal function using multivariable logistic regression models. The adjustment for case mix included the following covariates, selected a priori: age, Body Mass Index, preoperative serum creatinine, clinical T stage (cT1a vs. cT1b), PADUA score (continuous), warm ischemia time [14] (continuous), pathologic tumor size (continuous), and year of surgery (continuous). When 1 yr renal function was the outcome of interest, the models also included acute kidney injury. Within-surgeon clustering was incorporated into our analyses using the *cluster* option in Stata statistical software. There was no clustering by institution, as there is no plausible mechanism by which an institution could affect the learning curve and given that few of the surgeons moved between institutions. To produce a learning curve, we calculated the probability of each outcome of interest for an average patient, using the mean value for covariates.

We performed a number of sensitivity analyses to assess the robustness of our findings. Since clinical size and functional recovery might be correlated, we repeated the analyses only in patients with cT1a and—separately—with cT1b masses. Similarly, since year of surgery might be correlated with surgical experience, we repeated analyses after excluding this covariate. Finally, we assessed the hypothesis that the association between surgical experience and functional outcomes after surgery might differ for those surgeons who have achieved certain technical skills, as a result of the large number of cases performed in their career, by excluding patients treated by surgeons who had performed more than 400 procedures.

All statistical analyses were performed using Stata version 14.0 (StataCorp LP, College Station, TX, USA).

## 3. Results

### 3.1. Descriptive Characteristics of Surgeons Included in the Study

A total of 753 (19%) and 3258 (81%) patients underwent laparoscopic and robot-assisted partial nephrectomy, respectively. The distribution of surgeons by the total number of lifetime operations is shown in Table 1. Overall, 119 unique surgeons were included in the study. Of them, 37 (31%) and 55 (46%) contributed only to the laparoscopic and robotic learning curves, respectively, whereas 27 (23%) contributed to both approaches. Approximately half of the surgeons included in both groups had performed fewer than 50 cases in their career, whereas 6 (9%) and 14 (16%) surgeons had performed more than 250 laparoscopic and robotic procedures, respectively.

### 3.2. Surgical Experience and Functional Outcomes

#### 3.2.1. Laparoscopic Partial Nephrectomy

Descriptive characteristics of patients who underwent laparoscopic partial nephrectomy are summarized in Supplementary Table S1. Renal masses treated by more experienced surgeons were larger and more complex. The rate of off-clamp procedures was higher for more experienced surgeons (77% vs. 41% for cases performed by surgeons with ≥150 vs. <50 prior laparoscopic partial nephrectomies).

**Figure 1:**
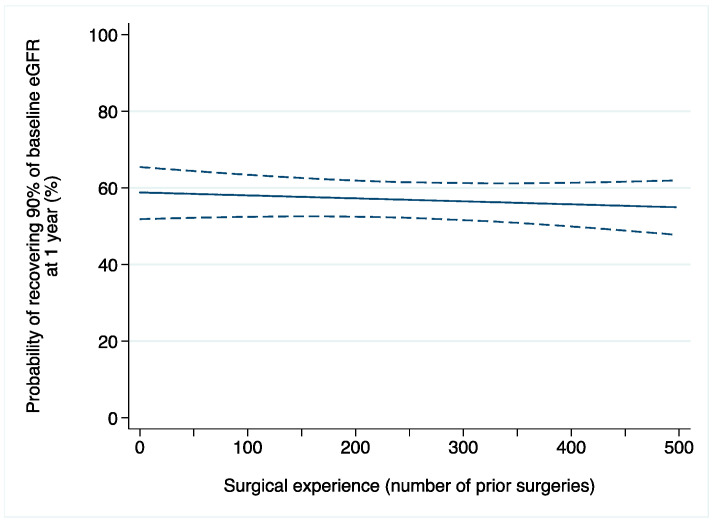
Probability of recovering at least 90% of baseline eGFR after laparoscopic partial nephrectomy in relation to surgical experience (number of prior operations). Dashed lines are 95% confidence interval.

A total of 61 (8%) patients developed AKI after surgery. Among those with available data on renal function one year after surgery (*n* = 444), 245 (55%) patients recovered at least 90% of their baseline eGFR. After adjusting for confounders, we did not find evidence of an association between surgical experience and acute kidney injury after laparoscopic partial nephrectomy (odds ratio [OR]: 0.9992; 95% confidence interval [CI]: 0.9963, 1.0022; *p* = 0.6; Table 2a). Similar results were found when 1-year renal function was the outcome of interest (OR: 0.9996; 95% CI: 0.9988, 1.0005; *p* = 0.5; Figure 1).

#### 3.2.2. Robot-Assisted Partial Nephrectomy

Supplementary Table S2 describes the baseline characteristics of patients treated with robot-assisted partial nephrectomy, stratified by increasing surgical experience. The baseline patient characteristics were quite similar across the surgeon groups. In comparison to less experienced surgeons, the rate of on-clamp procedures was higher in the more experienced group (81% vs. 76% for surgeons with ≥150 vs. <50 prior RAPNs; *p* = 0.015), whereas the duration of ischemia was shorter (median: 15 vs. 16 min; *p* < 0.0001).

**Figure 2:**
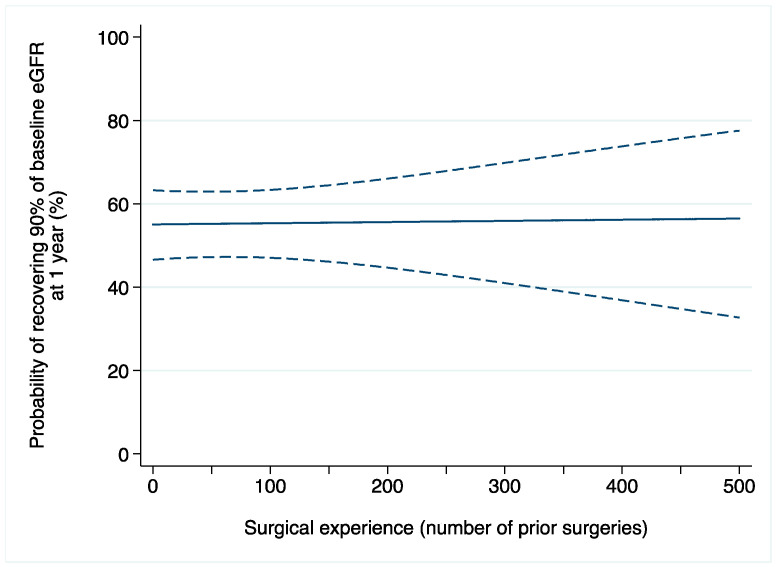
Probability of recovering at least 90% of baseline eGFR after robot-assisted partial nephrectomy over surgical experience (number of prior operations). Dashed lines are 95% confidence interval.

Acute kidney injury after surgery occurred in 369 (11%) patients. Among 1521 patients with available data on one-year renal function, 828 (54%) recovered at least 90% of their baseline eGFR. On multivariable logistic regression, the relationship between surgical experience and AKI after surgery was not statistically significant (OR: 1.0015; 95% CI: 0.9992, 1.0037; *p* = 0.2; Table 2b). We found similar results when the outcome of interest was renal function one year after surgery (OR: 1.0001; 95% CI: 0.9980, 1.0022; *p* = 0.9; Figure 2).

### 3.3. Sensitivity Analyses

We conducted a number of sensitivity analyses (Table 3). When we repeated the analyses on index patients with cT1a and—separately—cT1b renal masses, results were consistent with our main findings with only few exceptions. Similarly, results were unaltered after repeating the analyses with year of surgery as the covariate, both for laparoscopic and robotic partial nephrectomies. Finally, to specifically focus on the early phase of surgical learning, we restricted the analyses to patients operated on by surgeons with no more than 400 prior procedures performed on index patients. Similar to our main analyses, the relationship between surgical experience and functional outcomes after surgery did not reach statistical significance (all *p* > 0.05 for all outcomes of interests).

## 4. Discussion

### 4.1. Discussion

In this study, we investigated the relationship between surgical experience and functional outcomes after laparoscopic and robot-assisted partial nephrectomy. We did not find evidence that increasing the number of prior operations before an index patient’s surgery might affect functional recovery, both immediately after surgery and at long-term follow-up.

How surgical experience, the skills of the operating surgeon, and the outcomes of a particular operation are related is a complex and sophisticated matter [15,16]. One would expect that the more experienced the surgeon, the better the outcomes, but there is evidence that this is not always the case. We previously analyzed the outcomes of robot-assisted radical prostatectomy, showing that biochemical recurrence rates were not correlated with the experience of the operative surgeon, but rather, that they remained stable over time [9], a finding that was confirmed in a multi-institutional collaboration including 8101 patients treated by 46 surgeons [17]. In the context of partial nephrectomy, there are several other nuances to take into account. While prior evidence has shown that there is a learning curve for ischemia time and complications after surgery [2], our data strongly suggest that there is no such association when it comes to functional outcomes. This finding, consistent with prior data [18] and also with evidence on radical nephrectomies [19], may be explained by several reasons. For instance, it is agreeable that a complex operation such as partial nephrectomy might not be the first procedure a surgeon starts with in his/her career. In other words, it is plausible that a given surgeon might start performing partial nephrectomy once their surgical experience has reached a certain level, especially in the context of laparoscopic and/or robotic surgery. This, in turn, may lead surgeons to start their laparoscopic/robotic practice with partial nephrectomy once their surgical skills are adequate. This will allow them to achieve optimal outcomes in terms of functional recovery from their initial cases. While this is consistent with data on robot-assisted surgery [17], this study provides the first evidence on laparoscopic partial nephrectomy that aligns to such findings. There are many challenges with partial nephrectomy, and performing such a demanding operation laparoscopically remains in the hands of few surgeons. Once again, it is possible that they might have consolidated their skills in more straightforward operations (e.g., laparoscopic radical nephrectomy) before the start of their practice in partial nephrectomy.

Another possible explanation for our findings is related to the outcomes of interest of this study. Serum creatinine is surely a handy marker to assess renal function, and its widespread utilization makes it the reference parameter for observational studies. At the same time, its limits for functional follow-up after renal surgery are well known, especially in patients with a normal contralateral kidney [20]. In this context, the relative contribution of the operated kidney remains unknown when looking at serum creatinine. Some may argue that, to solve this issue, there might be some indirect indicators such as parenchymal mass reduction [21], the measure of functional loss in the operating kidney. Alternatively, a direct and precise assessment of the surgical footprint on renal function after surgery can be achieved with the use of renal scintigraphy and split renal function [22]. However, several factors—including cost and availability—limit the use of such methods in retrospective studies. That said, although implementations are welcome to improve our understanding of the detrimental effect of surgery on renal function, serum creatinine and its derivative are currently the measure of choice. It is utilized worldwide, and its fluctuations drive interventions such as replacement therapy. As a result, although we cannot exclude that our results might be affected by additional parameters, their cumulative effect would inevitably translate in the serum creatinine that we analyzed. Serum creatinine and its corollaries, especially acute kidney injury, are important markers for long-term recovery [5], and efforts should be made to limit surgically induced functional damage. In this regard, our data suggest that a surgeon’s experience might not play a relevant role in avoiding acute kidney injury as well as long-term functional recovery.

### 4.2. Limitations

Our study has several limitations. First, the retrospective study may be susceptible to selection bias and influenced by uncontrolled confounding factors. For instance, information on tumor location or on how surgeons were trained was not available in our database. In addition, retrospective studies often rely on existing records, which may not be representative of the broader patient population. For instance, patients who have complete records or who return for follow-up appointments might differ systematically from those who do not, leading to biased results. With respect to training, some may argue that our series included teaching institutions with trainees involved as console surgeons. Although we cannot completely rule out this factor, there is evidence that this is not problematic for surgical outcomes [23,24]. We also must acknowledge that our series might not emulate real-world settings. While less experienced surgeons might naturally gravitate away from complex patients, our findings should be interpreted in light of the aforementioned limitations. Another potential limitation concerns the multi-institutional nature of our dataset. The inclusion of a number of institutions from different regions and healthcare systems may have resulted in different surgical techniques or practices (e.g., patient selection and/or resection techniques) across centers. Similarly, our data on robot-assisted partial nephrectomy included only patients treated with similar robots. Given the introduction of new robotic systems into the market [25,26,27], it is possible that the learning curve might differ between platforms. Despite these limitations, our study represents the largest series investigating the learning curve for functional outcomes after partial nephrectomy in an adequately large number of surgeons and patients.

Our findings have implications for future research. The fact that novice surgeons were able to achieve functional outcomes similar to those of more experienced surgeons opens the discussion of what aspects of the operation underpin this finding. In the context of robot-assisted partial nephrectomy, performance metrics were recently developed and might be used as a reference to identify the key steps of the operation that translated into optimal functional outcomes [27]. Future investigations are awaited that extend this structured approach to laparoscopic partial nephrectomy.

Our results also highlight the need for standardized protocols to optimize patient outcomes regardless of surgical experience [28,29]. Establishing and adhering to such protocols can ensure consistency and high-quality care across various practitioners and institutions [30,31]. This approach can help minimize complications [32], reduce variability in ischemia times, and enhance overall peri-operative outcomes [30,31].

Finally, future studies should explore the impact of different surgical techniques on functional outcomes in a prospective manner. This approach would allow for the collection of high-quality, reliable data, helping to refine surgical practices and enhance patient care. Researchers should design well-structured, prospective studies that track patients from the point of surgery forward, systematically comparing the results of various surgical methods [32,33,34,35,36,37,38,39]. By doing so, they can identify which techniques yield the most favorable functional outcomes. To achieve this, it is crucial to encourage collaboration among surgical centers, enabling the sharing of data and insights across a larger patient population. Funding agencies and healthcare institutions should prioritize and support such research initiatives. Furthermore, surgeons and medical professionals should be actively involved in these studies to ensure the practical applicability of the findings. This concerted effort can lead to the development of evidence-based guidelines that optimize surgical techniques, ultimately improving patient recovery and quality of life [40,41,42,43,44,45,46,47,48].

## 5. Conclusions

In this large, multi-institutional project involving many surgeons, we did not find evidence of an association between functional recovery after partial nephrectomy—laparoscopic or robot-assisted—and the number of prior operations performed by the surgeon on index patient surgery. As such, our findings suggest that surgical experience may not significantly impact functional recovery following partial nephrectomy. While potential explanations include the use of serum creatinine as a marker of functional recovery in patients with two kidneys, it is important to investigate the key steps of the procedure that allowed novices to have comparable outcomes as compared to more experienced surgeons. These represent the main focus for future educational efforts and should be taken into account during the training and proctoring of surgeons who start their practice in laparoscopic or robot-assisted partial nephrectomy.

## Figures and Tables

**Table 1:** Distribution of number of surgeons and patients according to (A) total lifetime number of robotic procedures performed and (B) median caseload/year per surgeon.

	Laparoscopic		Robotic	
**Total Lifetime Number of Partial Nephrectomies Performed (A)**	**Surgeons,** ***n* (%)**	**Patients,** ***n* (%)**	**Surgeons, *n* (%)**	**Patients,** ***n* (%)**
0–24	30 (46)	131 (17)	39 (45)	114 (3)
25–49	8 (13)	120 (16)	8 (9)	121 (4)
50–99	9 (13)	22 (3)	10 (15)	225 (7)
100–249	11 (17)	206 (27)	11 (15)	683 (21)
250–499	4 (6)	212 (28)	9 (9)	771 (24)
≥500	2 (3)	62 (8)	5 (7)	1344 (41)
Total	64 (100)	753 (100)	82 (100)	3258 (100)
**Median annual number of partial nephrectomies performed (B)**				
≤10	50 (79)	377 (50)	45 (55)	215 (7)
11–29	11 (16)	247 (33)	18 (22)	500 (16)
30–99	2 (3)	62 (8)	18 (22)	2272 (68)
100–149	1 (2)	67 (9)	1 (1)	271 (9)
≥150	-	-	-	-
Total	64 (100)	753 (100)	82 (100)	3258 (100)

**Table 2:** Multivariable analyses assessing the association between surgical experience (number of prior cases) and functional outcomes after (a) laparoscopic and (b) robot-assisted partial nephrectomy. All odds ratios and 95% confidence intervals refer to surgical experience included as continuous, non-linear term. CI: confidence interval. eGFR: estimated glomerular filtration rate.

**a—Laparoscopic (*n* = 753)**			
**Outcome**	**Odds Ratio**	**95% CI**	***p***
**Acute kidney injury**	0.9992	0.9963, 1.0022	0.6
**Recovery of 90% of preoperative eGFR at 1 year follow-up**	0.9996	0.9988, 1.0005	0.5
**b—Robotic (*n* = 3258)**			
**Outcome**	**Odds ratio**	**95% CI**	***p***
**Acute kidney injury**	1.0015	0.9992, 1.0037	0.2
**Recovery of 90% of preoperative eGFR at 1 year follow-up**	1.0001	0.9980, 1.0022	0.9

Models adjusted for age, Body Mass Index, preoperative serum creatinine, clinical T stage, PADUA score, warm ischemia time, pathologic tumor size, and year of surgery. When functional outcomes at one year were the outcomes of interest, the adjustment for case mix also included acute kidney injury.

**Table 3:** Sensitivity analyses assessing the association between surgical experience (the number of prior cases) and functional outcomes after (a) laparoscopic and (b) robot-assisted partial nephrectomy. All odds ratios and 95% confidence intervals refer to surgical experience included as continuous, non-linear term. OR: odds ratio. CI: confidence interval. eGFR: estimated glomerular filtration rate.

**a—Laparoscopic**				
	**Acute Kidney Injury**		**Recovery of 90% of Preoperative eGFR at 1-Year Follow-Up**	
	**OR (95% CI)**	***p***	**OR (95% CI)**	***p***
Main analyses	1.00 (1.00, 1.00)	0.5	1.00 (1.00, 1.00)	0.6
According to cT stage				
Only cT1a	Non-linear	0.012	1.00 (1.00, 1.00)	0.8
Only cT1b	1.00 (1.00, 1.00)	0.9	1.00 (1.00, 1.00)	0.6
Excluding covariates: Year of surgery	1.00 (1.00, 1.00)	0.7	1.00 (1.00, 1.00)	0.3
Exclude patients whose surgeon completed >400 prior procedures	1.00 (1.00, 1.00)	0.6	1.00 (1.00, 1.00)	0.5
**b—Robotic**				
	**Acute Kidney Injury**		**Recovery of 90% of Preoperative eGFR at 1-Year Follow-Up**	
	**OR (95% CI)**	***p***	**OR (95% CI)**	***p***
Main analyses	1.00 (1.00, 1.00)	0.2	1.00 (1.00, 1.00)	0.9
According to cT stage				
Only cT1a	1.00 (1.00, 1.00)	0.10	1.00 (1.00, 1.00)	0.4
Only cT1b	1.00 (1.00, 1.00)	0.5	Non-linear	0.040
Excluding covariates: Year of surgery	1.00 (1.00, 1.00)	0.12	1.00 (1.00, 1.00)	0.4
Exclude patients whose surgeon completed >400 prior procedures	1.00 (1.00, 1.00)	0.4	1.00 (1.00, 1.00)	0.7

Models adjusted for age, Body Mass Index, preoperative serum creatinine, clinical T stage, PADUA score, warm ischemia time, pathologic tumor size, and year of surgery. When functional outcomes at one year were the outcomes of interest, the adjustment for case mix also included acute kidney injury.

## Data Availability

The data presented in this study are available on request from the corresponding author due to privacy reasons.

## Supplementary Materials

The following supporting information can be downloaded at: https://www.mdpi.com/article/10.3390/jcm13196016/s1, Figure S1: Inclusion/Exclusion criteria and methods flowchart.; Table S1: Descriptive characteristics of 753 patients treated with laparoscopic partial nephrectomy by increasing surgical experience of the surgeon at the time of index patient’s operation. All numbers are medians (interquartile range) and frequencies (percentages). eGFR: estimated glomerular filtration rate.; Table S2: Descriptive characteristics of 3258 patients treated with robot-assisted partial nephrectomy by increasing surgical experience of the surgeon at the time of index patient’s operation. All numbers are medians (interquartile range) and frequencies (percentages). eGFR: estimated glomerular filtration rate.
