# Supplementary material for: Surgical Experience and Functional Outcomes after Laparoscopic and Robot-Assisted Partial Nephrectomy: Results from a Multi-Institutional Collaboration
Source: J Clin Med. 2024 Oct 9;13(19):6016. doi: 10.3390/jcm13196016 (PMC11477761; doi:10.3390/jcm13196016)
Supplement: Supplementary file 1 [file jcm-13-06016-s001.zip › jcm-3079239-supplementary.pdf]

Supplementary Figure S1. Inclusion/Exclusion criteria and methods flowchart.

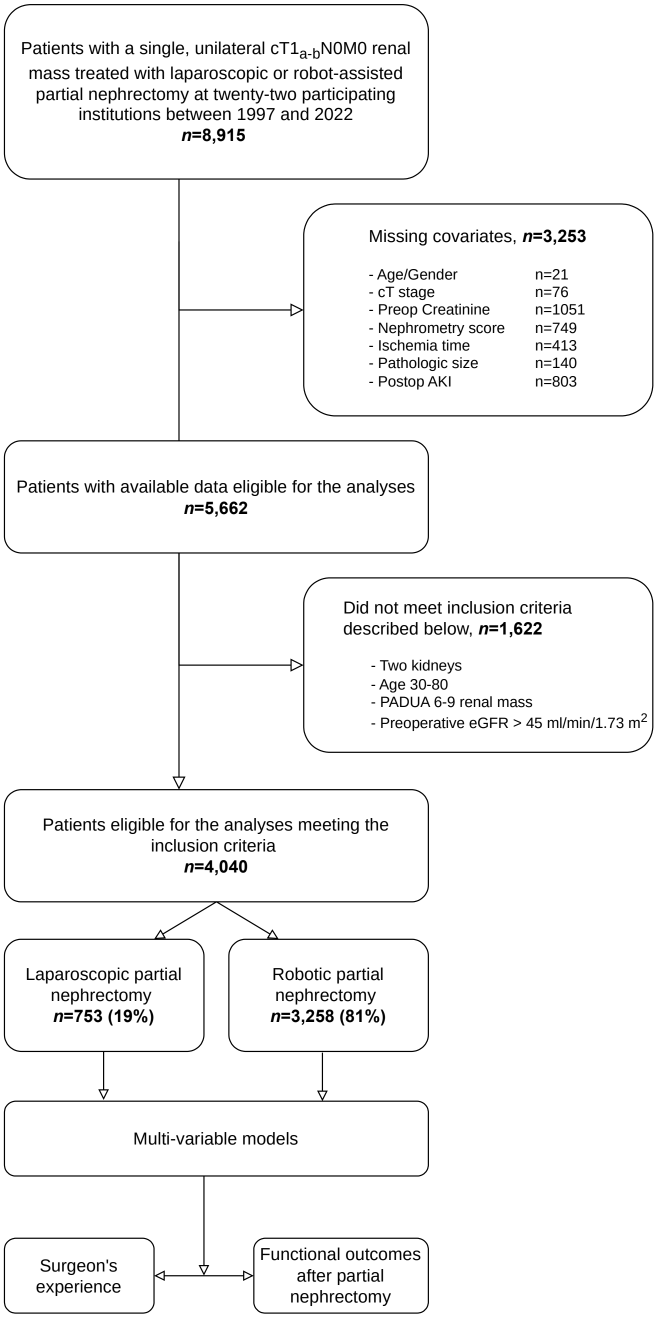

Supplementary Table S1. Descriptive characteristics of 753 patients treated with laparoscopic partial nephrectomy by increasing surgical experience of the surgeon at the time of index patient's operation. All numbers are medians (interquartile range) and frequencies (percentages). eGFR: estimated glomerular filtration rate.

	**Overall (N=753; 100%)**	**0-49 (N=313; 42%)**	**50-149 (N=189; 25%)**	**150+ (N=251; 33%)**	**p**
**Age**, years	62 (54, 70)	62 (53, 70)	64 (56, 71)	63 (54, 70)	0.2
**Gender**, Male	532 (71%)	231 (74%)	130 (69%)	171 (68%)	0.3
**Body Mass Index**	26 (24, 29)	26 (24, 29)	26 (24, 28)	26 (24, 28)	0.015
**Preoperative creatinine**, mg/dl	0.9 (0.8, 1.0)	0.9 (0.8, 1.0)	0.9 (0.8, 1.0)	0.9 (0.8, 1.0)	0.4
**cT stage**					
T1a	629 (84%)	273 (87%)	158 (84%)	198 (79%)	0.030
T1b	124 (16%)	40 (13%)	31 (16%)	53 (21%)	
**Clinical size**, cm	2.7 (2.0, 3.5)	2.7 (2.0, 3.4)	2.5 (2.0, 3.4)	3.0 (2.4, 4.0)	0.001
**Lesion side**, Left	362 (48%)	143 (46%)	95 (50%)	124 (49%)	0.5
**PADUA score**					
6-7	487 (65%)	218 (70%)	123 (65%)	146 (58%)	0.018
8-9	266 (35%)	95 (30%)	66 (35%)	105 (42%)	
**Ischemia type**					
No ischemia	435 (58%)	127 (41%)	115 (61%)	193 (77%)	<0.0001
Warm	318 (42%)	186 (59%)	74 (39%)	58 (23%)	
**Warm ischemia time**, minutes	17 (14, 23)	16 (13, 22)	19 (15, 24)	17 (14, 23)	0.15
**Estimated blood loss**	100 (50, 200)	100 (50, 200)	150 (100, 250)	100 (50, 200)	0.002
**Pathology**					
Benign	143 (19%)	54 (17%)	43 (23%)	46 (18%)	0.3
Malignant	591 (78%)	259 (83%)	144 (76%)	188 (75%)	
Unknown	19 (3%)	-	2 (1%)	17 (7%)	
**Pathologic stage**					
Benign	143 (19%)	54 (17%)	43 (23%)	46 (18%)	<0.0001
pT1a	508 (67%)	220 (70%)	124 (66%)	164 (65%)	
pT1b	77 (10%)	28 (9%)	10 (5%)	39 (16%)	
pT2a	7 (1%)	1 (1%)	6 (3%)	-	
pT3+	18 (3%)	10 (3%)	6 (3%)	2 (1%)	
**Pathologic size**, cm	3.0 (2.0, 4.2)	2.7 (2.0, 4.0)	2.5 (2.0, 3.5)	3.5 (2.5, 15.0)	<0.0001
**Positive surgical margins**	62 (8%)	39 (12%)	12 (6%)	11 (4%)	0.002
Unknown	12 (2%)	3 (1%)	-	9 (3%)	
**1-yr renal function***					
Creatinine, mg/dl	1.0 (0.8, 1.1)	1.0 (0.9, 1.1)	0.9 (0.9, 1.1)	1.0 (0.8, 1.1)	0.7
eGFR, ml/min/1.73m^2	79 (64, 92)	80 (65, 92)	80 (69, 92)	78 (60, 91)	0.2

* Available for 444 patients.

Supplementary Table S2. Descriptive characteristics of 3258 patients treated with robot-assisted partial nephrectomy by increasing surgical experience of the surgeon at the time of index patient's operation. All numbers are medians (interquartile range) and frequencies (percentages). eGFR: estimated glomerular filtration rate.

	Overall (N=3258; 100%)	0=49 (N=636; 20%)	50-149 (N=856; 26%)	150+ (N=1766; 54%)	p
**Age**, years	62 (53, 70)	62 (52, 70)	62 (54, 70)	62 (54, 69)	0.8
**Gender**, Male	2152 (66%)	410 (64%)	576 (67%)	1166 (66%)	0.5
**Body Mass Index**	26 (24, 30)	26 (24, 29)	26 (24, 29)	26 (24, 30)	0.015
**Preoperative creatinine**, mg/dl	0.9 (0.8, 1.0)	0.9 (0.8, 1.0)	0.9 (0.8, 1.0)	0.9 (0.8, 1.0)	0.028
**cT stage**					
T1a	2530 (78%)	498 (78%)	675 (79%)	1357 (77%)	0.5
T1b	728 (22%)	138 (22%)	181 (21%)	409 (23%)	
**Clinical size**, cm	3.0 (2.2, 4.0)	3.0 (2.2, 4.0)	3.0 (2.1, 4.0)	3.0 (2.1, 4.0)	0.3
**Lesion side**, Left	1639 (50%)	323 (51%)	439 (51%)	877 (50%)	0.7
Unknown	4 (<1%)	1 (<1%)	-	3 (<1%)	
**PADUA score**					
6-7	1733 (53%)	351 (55%)	469 (55%)	913 (52%)	0.2
8-9	1525 (47%)	285 (45%)	387 (45%)	853 (48%)	
**Ischemia type**					
No ischemia	650 (20%)	153 (24%)	159 (19%)	338 (19%)	0.015
Warm	2608 (80%)	483 (76%)	697 (81%)	1428 (81%)	
**Warm ischemia time**, minutes	15 (11, 19)	16 (12, 21)	15 (11, 19)	15 (10, 19)	<0.0001
**Estimated blood loss**	150 (75, 300)	150 (85, 300)	200 (100, 346)	150 (50, 300)	<0.0001
**Pathology**					
Benign		111 (17%)	157 (18%)	312 (18%)	0.8
Malignant		379 (60%)	587 (69%)	1110 (63%)	
Unknown		146 (23%)	112 (13%)	344 (19%)	
**Pathologic stage**					
Benign	580 (18%)	111 (17%)	157 (18%)	312 (18%)	0.6
pT1a	2002 (61%)	386 (61%)	519 (61%)	1097 (61%)	
pT1b	460 (14%)	100 (16%)	116 (14%)	244 (14%)	
pT2a	18 (<1%)	-	5 (1%)	13 (1%)	
pT2b	7 (<1%)	1 (1%)	3 (1%)	3 (1%)	
pT3+	160 (5%)	27 (3%)	45 (4%)	88 (4%)	
Unknown	31 (1%)	11 (2%)	11 (1%)	9 (1%)	
**Pathologic size**, cm	2.9 (2.0, 3.9)	3.0 (2.2, 4.0)	2.8 (2.0, 3.9)	2.9 (2.0, 3.9)	0.085
**Positive surgical margins**	276 (8%)	44 (7%)	78 (9%)	154 (9%)	0.3
Unknown	83 (2%)	14 (2%)	21 (2%)	48 (3%)	
**1-yr renal function***					
Creatinine, mg/dl	1.0 (0.9, 1.3)	1.0 (0.9, 1.4)	1.0 (0.9, 1.3)	1.0 (0.9, 1.2)	0.094
eGFR, ml/min/1.73m^2	73 (54, 87)	70 (48, 85)	72 (55, 88)	74 (58, 88)	0.12

* Available for 1521 patients.
